# Functionalized Chitosan Nanomaterials: A Jammer for Quorum Sensing

**DOI:** 10.3390/polym13152533

**Published:** 2021-07-30

**Authors:** Moupriya Nag, Dibyajit Lahiri, Dipro Mukherjee, Ritwik Banerjee, Sayantani Garai, Tanmay Sarkar, Sujay Ghosh, Ankita Dey, Sougata Ghosh, Smaranika Pattnaik, Hisham Atan Edinur, Zulhisyam Abdul Kari, Siddhartha Pati, Rina Rani Ray

**Affiliations:** 1 Department of Biotechnology, University of Engineering & Management, Kolkata 700160, India; moupriya.nag@uem.edu.in (M.N.); dibyajit.lahiri@uem.edu.in (D.L.); dipmukherjee23@gmail.com (D.M.); ritwik2809@gmail.com (R.B.); sayantani0717@gmail.com (S.G.); 2 Department of Food Technology and Bio-Chemical Engineering, Jadavpur University, Kolkata 700032, India; tanmay@wbscte.ac.in; 3 Malda Polytechnic, West Bengal State Council of Technical Education, Government of West Bengal, Malda 732102, India; 4 AMH Energy Pvt. Ltd., Kolkata 700039, India; sujayxghosh@gmail.com; 5 Department of Biotechnology, Maulana Abul Kalam Azad University of Technology, Haringhata 741249, India; ankita.dey16061996@gmail.com; 6 Department of Microbiology, School of Science, RK. University, Rajkot 360020, India; ghoshsibb@gmail.com; 7 Department of Biotechnology and Bioinformatics, Sambalpur University, Odishsa 768001, India; smaranika2010@gmail.com; 8 School of Health Sciences, Universiti Sains Malaysia, Health Campus, Kubang Kerian, Kelantan 16150, Malaysia; 9 Faculty of Agro Based Industry, Universiti Malaysia Kelantan, Kota Bharu 17600, Malaysia; 10 Centre of Excellence, Khallikote University, Berhampur 761008, India; 11 SIAN Institute, Association for Biodiversity Conservation and Research (ABC), Odisha 756001, India

**Keywords:** antibiofilm, chitosan, nanomaterial, quorum quenching, quorum sensing

## Abstract

The biggest challenge in the present-day healthcare scenario is the rapid emergence and spread of antimicrobial resistance due to the rampant use of antibiotics in daily therapeutics. Such drug resistance is associated with the enhancement of microbial virulence and the acquisition of the ability to evade the host’s immune response under the shelter of a biofilm. Quorum sensing (QS) is the mechanism by which the microbial colonies in a biofilm modulate and intercept communication without direct interaction. Hence, the eradication of biofilms through hindering this communication will lead to the successful management of drug resistance and may be a novel target for antimicrobial chemotherapy. Chitosan shows microbicidal activities by acting electrostatically with its positively charged amino groups, which interact with anionic moieties on microbial species, causing enhanced membrane permeability and eventual cell death. Therefore, nanoparticles (NPs) prepared with chitosan possess a positive surface charge and mucoadhesive properties that can adhere to microbial mucus membranes and release their drug load in a constant release manner. As the success in therapeutics depends on the targeted delivery of drugs, chitosan nanomaterial, which displays low toxicity, can be safely used for eradicating a biofilm through attenuating the quorum sensing (QS). Since the anti-biofilm potential of chitosan and its nano-derivatives are reported for various microorganisms, these can be used as attractive tools for combating chronic infections and for the preparation of functionalized nanomaterials for different medical devices, such as orthodontic appliances. This mini-review focuses on the mechanism of the downregulation of quorum sensing using functionalized chitosan nanomaterials and the future prospects of its applications.

## 1. Introduction

The last decade has seen a marked increase in the development of multi-drug-resistant pathogenic organisms that have brought about significant threats for the health sector. Numerous alternative approaches are being taken to check the pathogenesis of these antibiotic-resistant microbes and strategies are being adopted to minimize their virulence [1,2]. The chronic-infection-causing recalcitrant microbes usually reside in the protective shield of their biofilm, which is actually a syntrophic association of microbes. Hence, exploration of the natural ways for biofilm eradication and innovations for biotechnological approaches to enhance their antibiofilm activity becomes a new and booming stream of research.

Both microbes alone and the biofilm formed by them attach themselves to specific surfaces. The biofilm-associated cells are especially capable of forming an extracellular polymeric substance matrix (EPS), which can maintain decreased growth rates and allow for up- and down regulation of some specific genes [3,4]. The EPS matrix possesses a definite construction pattern and creates an optimal condition that allows the microbes to exchange genetic contents between the cells [5]. Moreover, the biofilm-forming cells undergo cell-to-cell communication via the process of quorum sensing (QS), by which they control the expression of genetic components in response to continuous changes in the density of the cell population [6]. QS is accomplished by various types of extracellular communication materials called autoinducers (AIs) [7], which are the chemical signaling molecules that are synthesized and released by these cells [8].

Since QS plays a key role in bacterial infection and bacterial survival, eradication of the biofilm through the denaturation of the AI molecules will ensure the prevention of biofilm-associated infection. Several novel antibiofilm agents were developed for interfering with the QS cascade and thereby inhibiting the formation of biofilms [9,10].

However, such interruption in cellular communication can be done via the mechanism of quorum quenching (QQ), which involves the process of disrupting the QS cascade [7]. The molecular mechanism of QQ includes the cleavage of QS signals, competitive inhibition, and acting against the major targets of QS, thereby bringing about hindrance in the maturation of biofilm.

Present-day nanomaterials are largely used as alternate therapeutics due to their large surface-area-to-volume ratio and extensive reactivity, resulting in the development of the new field of “nanomedicines” [11,12,13]. The enhancement in the development of antimicrobial resistance has resulted in researchers thinking about ways to provide alternate therapeutics [14]. A fascinating thing about nanomaterials is that their efficacies are largely dependent on the shape and size of the nanostructural contents of the nanomaterials and these properties can usually be distinguished well from the bulk traditional material, which possesses the appearance of a continuous material [15]. This is why these nanomaterials create huge interest regarding their applications in different types of research and development fields related to biotechnology, biology, chemistry, biophysics, and many others [16].

In order to surpass the existing drawbacks of nanomaterials, there has recently been development of innovative functionalized nanomaterials with potential applications in various healthcare domains. For covering the nanoparticle core surface with a capping agent [17], the surface of nanomaterials can be functionalized using either a covalent modification strategy via a standard organic synthesis procedure or using a noncovalent modification complexation, adsorption process, or grafting strategy [18].

Several researchers are trying to use functionalized nanoparticles to resist biofilm formation, where gold particles (GNPs) functionalized with enzyme proteinase K were found to be effective against *Pseudomonas fluorescens* biofilms [19]; silica nanoparticles functionalized with either peppermint oil (P-Cap) alone or in combination with cinnamaldehyde (CP-Cap) were found to halt the complex biofilm formation of *Pseudomonas aeruginosa, E. coli DH5, S. aureus,* and *Enterobacter cloacae* [20]; and amine-, carboxylate-, and isocyanate-functionalized superparamagnetic-iron oxide nanoparticles (IONs) against *S. aureus* biofilms are noteworthy [21]. Gold-nanoparticle-functionalized liposomes containing tobramycin could decrease biofilm biomass by approximately 1.5 times as compared to untreated liposomes containing tobramycin only [22]. A catheter surface that was functionalized with MgF(2) nanoparticles (NPs) effectively removed the biofilm from it [23]. Gold nanoparticles (GNPs) and gold nanocomposites functionalized with antimicrobial peptide Pediocin AcH and *Listeria* adhesion protein (LAP) (GNP–Pediocin–LAP) were successfully used to remove biofilms of *Listeria* sp. [24].

Metals and metal oxide nanoparticles (NPs) were associated with inorganic and organic supports to improve their antibacterial activity and stability. These nanomaterials can be triggered by various mechanisms (such as changes in pH, light, magnetic fields, and the presence of bacterial enzymes); additionally, they can improve antibacterial efficacy and reduce side effects and microbial resistance [25].

Apart from functionalized metal nanoparticles, functionalized chitosan nanoparticles were also found to enhance photodynamic therapy and thereby have a 100% bactericidal activity against *Enterococcus faecalis* [26]. Actually, chitosan is often preferred for use as the component of nanomaterials due to its biocompatibility, verified low toxicity verified, bio- and muco-adhesivity, biodegradability, etc. [27,28,29,30].

This review discusses the functionalization of chitosan nanoparticle and elucidation of its efficacy in interrupting/blocking the quorum sensing (QS) signal to halt biofilm formation.

## 2. Quorum Sensing in Biofilm-Associated Microbes

QS, being the key event behind biofilm formation, is the main target for blocking to achieve an antibiofilm effect. Apart from biofilm formation, QS regulates multiple processes that involve sporulation, bioluminescence, the production of various types of virulence factors, antibiotic biosynthesis, and the formation of biofilms [31,32]. The mechanism of QS in Gram-negative bacteria (Table 1) takes place via LuxI/LuxR type systems, which play an important role in the production of AIs, the signalling molecules [33].

## 3. Chitosan Nanoparticles

A biofilm matrix acting as a scaffold provides a protective covering for sessile bacteria, making them drug resistant [50]. Hence, a more effective drug delivery system needs to be applied that can target both the biofilm matrix and the embedded sessile bacterial cells. Chitosan and its derivatives, with their acclaimed biofilm inhibiting property, may be used but in a more precise manner to halt the quorum sensing.

Nanoparticles, with atomic dimensions of 10Å to 100Å [51] were shown to be quite effective for drug delivery. Despite a few drawbacks, including poor absorption and dissolution rate with reduced bioavailability, using nanoparticles is a much safer method, as these microscopic particles act as nanocarriers, encasing high drug payloads and provide more targeted action with a controlled release.

Chitin, a natural polymer of β-(1,4)-N-acetyl-D-glucosamine, turns into chitosan, a polysaccharide composed of N-acetylglucosamine and D-glucosamine units [52], upon deacetylation in the presence of an alkali. Due to its cationic nature, biodegradability, compatibility, and nontoxicity, chitosan is used extensively by nano-biomedical researchers [53,54,55] for the delivery and controlled release of biomolecules, such as proteins, peptides, enzymes, genes, vaccines, and small drug molecules [56] via various delivery routes, including oral, buccal, vaginal, and pulmonary. Chitosan NPs are also used as vaccine adjuvants due to the mucoadhesive properties of chit, which can stimulate the cells of the immune system [57]. Some of the important properties of chitosan that have led to its wide range of applications in various fields (such as NPs) include mucoadhesion (as shown by trimethyl chitosan and carboxymethyl chitosan) [58]; controlled drug release, which enhances its effectiveness for drug delivery [59]; permeation enhancement, as shown by trimethyl chitosan [60]; antibacterial activity; no cytotoxicity; biocompatibility; and biodegradability. These properties are incredibly advantageous for the advancement of biocompatible and biodegradable medication conveyance frameworks [61,62].

Since its first emergence in the mid-1990s, the chitosan nanoparticle (ChNP) has been used for drug delivery [63]. The property that is responsible for the success of ChNPs in drug delivery is its ability to bind with negatively charged anions to form beads. However, beads larger than approximately 2mm generally hinder this process [64]. The discovery of the ChNPs involves various ‘bottom-up’ or ‘top-down’ approaches, or a synergistic combination of both techniques. However, among the regular ‘bottom-up’ methods, the most popular ones are ionotropic gelation and the polyelectrolyte complex method [65] due to their straightforwardness and non-requirement of high shear power and natural solvents [66], unlike the ‘top-down’ methods of milling, ultrasonication, and high-pressure homogenization [62,67].Irrespective of the methodology adopted for their preparation, the ChNPs are regularly used for drug delivery to combat several diseases with appreciable efficacy (Table 2). Although the precise mode of antimicrobial action is not determined completely, it was proposed that the molecular structure of chitosan is imperative for its antimicrobial activities. The antibacterial potential of chitosan is strongly influenced by several factors, such as its type, degree of polymerization, and physicochemical properties.

### 3.1. Preparation of Chitosan Nanoparticles (ChNPs)

**Figure 1:**
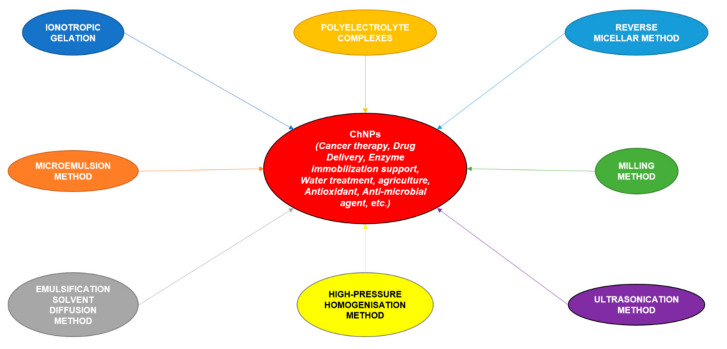
Schematic representation of the various methods employed for ChNP synthesis.

ChNPs were first prepared in the mid-1990s by scientist Ohya and his colleagues, who used the method of emulsifying and cross-linkage for the site-specific intravenous delivery of the anti-cancer drug (5-fluorouracil) [63]. The property that is responsible for the success of ChNPs in drug delivery is its ability to bind with negatively charged anions to form beads. However, beads larger than approximately 2 mm generally hinder this process [64]. The discovery of the ChNPs involves various ‘bottom-up’ or ‘top-down’ approaches, or a synergistic combination of both techniques. Currently, five methods are widely used for the synthesis of ChNPs. The ‘bottom-up’ methods include ionotropic gelation, microemulsion method, emulsification solvent diffusion/evaporation method, reverse micellar method and polyelectrolyte complex method [65]. Among these aforesaid methods, the most popular ones are ionotropic gelation and polyelectrolyte complex method. These techniques are straightforward and do not require high shear power or utilize natural solvents [66]. On the other hand, the ‘top-down’ methods are milling, ultrasonication, and high-pressure homogenization [62,67]. Figure 1 is a schematic representation of the various methods that are employed for the synthesis of ChNPs.

#### 3.1.1. Ionotropic Gelation

This method makes use of the crosslinking of the electrostatic bond between the amine groups of the chitosan to a polyanionic crosslinker, such as tripolyphosphate (TPP). The ionotropic gelation method for chitosan nanoparticle synthesis was first investigated by Calvo et al. in 1997 [68]. Ch can be dissolved in the presence or absence of a stabilizing agent (such as poloxamer) in acetic acid. This aqueous acidic solution of chitosan is then added dropwise to a TPP solution, with continuous and steady mechanical stirring at room temperature. Given that TPP is anionic, it will spontaneously crosslink with chitosan, forming chitosan–TPP NPs. This resulting product can successfully trap drug molecules and is able to carry them to a target. Hence, these nanocarriers were later developed into suitable drug delivery mechanisms. The dimensions and the charge on the surface can be changed by altering the chitosan-stabilizer ratio [69]. Alterations in the chitosan concentration and the polymer-to-polyanion ratio and an increase in the particle condensation and dimensions are also observed [70]. It was also reported that stability was increased when the NPs were added to a saline solution. This is because when monovalent sodium chloride salt is added to the solvent, there is an electrostatic repulsion between it and the amino group (positively charged) on the chitosanic backbone. This process decreases the particle size of the nanoparticles in the solution and increases the flexibility of the polymer chains, which helps to increase their stability [71].

In 2018, Furtado et al. reported the synthesis of chitosan and sodium fluoride (Ch-NaF) nanoparticles using this method [72]. This method is generally considered safe since it removes the dangers and toxicity that are associated with the use of organic solvents; it is also considered simple and easy with the use of an aqueous medium [62]. However, the nanoparticles synthesized using this method of ionotropic gelation generally have low mechanical strength [73].

#### 3.1.2. Microemulsion Method

This method was first reported by De et al. in 1999 [74] and involves the use of four components: polymer, surfactant, and a crosslinker. According to this method, a surfactant was dissolved in an organic solvent (n-hexane and toluene) and chitosan in an acetic acid solution. Glutaraldehyde (commonly used crosslinker for this method) was added to the surfactant/hexane and toluene mixture at room temperature, with continuous and steady mechanical stirring applied overnight, which completes the crosslinking process between the amino group of chitosan and glutaraldehyde, which acts as a crosslinker [75]. Subsequently, nanoparticles are formed. The main mechanism behind this crosslinking process is the Schiff reaction and comprises the mixture of the two solutions in the solvents, followed by the removal of excess surfactant as a precipitate with calcium chloride (CaCl_2_); then, the precipitate was removed using centrifugation to yield the desired polymer crosslinker nanoparticles [76].

However, the major drawback of this method lies in the use of the antigenic crosslinker agent glutaraldehyde [69]. Additionally, the integration of the peptide molecules to the synthesized nanoparticles may be hindered by the crosslinking and, thus, is not possible [68].

#### 3.1.3. Emulsification Solvent Diffusion Method

This improved method employed PGLA was first reported in 2002 [77] and is based on a method developed by Niwa et al. in 1993 [78]. A biological phase is injected into a chitosan solution with a poloxamer to enhance the stability of the solution; then mechanical stirring is applied, followed by a high-pressure homogenization technique to yield an emulsified mixture, which is diluted with water in the subsequent steps. The water used for dilution diffuses into the organic layer, which helps in the formation of the nanoparticles. The use of high shear forces during the synthesis of the nanoparticles and the involvement of organic solvents are the major limitations of this method [69].

#### 3.1.4. Polyelectrolyte Complexation (PEC) Method

Unlike any other method listed above, this method of chitosan nanoparticle synthesis involves the bond between the positively charged amino groups of chitosan and the anionic carboxylic group of dextran or alginate groups of dextran sulfate, which finally results in the neutralization of the charges. The self-assembly of the polyelectrolyte complexes occurs due to the charge neutralization via the addition of acidic chitosan solution into the anionic dextran solution with continuous mechanical stirring at room temperature [79,80]. Nanoparticles that carry insulin molecules to their target site were reported by the scientists to be manufactured by the process of alginate ionotropic pre-gelation and then subsequently by polyelectrolytic complexation with chitosan for diabetic patients [81]. Chitosan nanoparticles incorporated with gum are manufactured using the PEC method for bone regeneration therapy [82].

#### 3.1.5. Reverse Micellar Method

The most significant feature of this process is, unlike the other methods, the absence of both a crosslinker and poisonous natural solvents. Furthermore, ultrafine nanoparticles inside a restricted size reach can be acquired with this technique. This process can be described as the application of chitosan into the natural solvent containing the surfactant while the mixture is continuously agitated via mechanical stirring overnight to form the reverse micelles [83]. This method for the synthesis of ChNPs was first reported by a group of scientists in 2008 [84].

According to the recent trends and literature, among all the methods employed for the production of nanoparticles using chitosan, the most preferred and popular method is ionotropic gelation, which involves the ionic interaction between the cationic chitosan amine group and the anionic polymeric materials. This charge-based association of adversely charged drug payloads isa more manageable ionic gelation technique, bringing about a high drug encapsulation efficiency and optimal drug release such that the entrapment efficiency fluctuates between 0.3 to 0.98 and that of the drug release fluctuates between 0.4 and 0.8 [85]. The second-most common technique is polyelectrolyte complexation, which includes the crosslinking of chitosan with the molecules of the drug and results in a slowed drug discharge rate. Based on the literature evidence, the methods that are used to a lesser extent include solvent evaporation, coprecipitation, and emulsion droplet methods due to their poor entrapment efficiency and poor cargo release profile [62].

**Table 2:** ChNPs: preparation methods for various diseases, drugs, efficiencies, and advantages.

Method of Preparation	Diseases	Drug in ChNPs	Efficiency	Advantages	Reference
Ionotropic gelation	Bladder cancer	Chitosan–hyaluronic acid dialdehyde NPs (for CD44-targeted siRNA delivery)	LE ≥ 0.95	Cytotoxicity is reduced	[86]
	Migraine	Sumatriptan succinate	EE = 0.60	Targeted specific drug delivery	[87]
	*S. pneumoniae* infections	Cpl-1-loaded ChNPs	EE = 0.60	Enhanced bioavailability of the drug and in vivo half-life; chitosan biocompatibility for drug delivery	[65]
	Immuno-therapy	CpG oligodeoxynucleotide	EE = 0.90–0.97	Better immune-stimulation, cell uptake, and binding abilities	[88]
	Antimicrobial activity against MRSA	N′-((5-nitrofuran-2-yl) methylene)-2-benzohydrazide [(CH-5-NFB-NP)]	EE = 0.45	Antibacterial property increased; effective against multi-drug-resistant strains; easy production method	[89]
	Acne	Clindamycin	EE = 0.42	Better drug distribution; specific target delivery	[90]
	Administration of antioxidant peptides	Goby fish protein hydrolysate	EE = 0.61	Better thermal stability and antioxidant properties; controlled diffusion mechanism	[91]
	Hyperlipidemia	Sodium alginate entrapping rosuvastatin	-	Controlled drug release	[92]
	Phylloquinone induced prolonged blood circulation time	VK1	EE = 0.79	Constant release of vitamin K1; circulation time of RBC-hitchhiking chitosan NPs greater than regular NPs	[93]
	Polycystic kidney	Metformin	LE = 0.33	Enhanced bioavailability; lesser side effects in other parts of the body; better pharmaceutical efficacy	[94]
Polyelectrolyte complexation (PEC)	Cancer	Amygdalin entrapped by alginate	EE = 0.90	Stable release of the drug; low toxicity to cells	[95]
	Gene therapy	siRNA	-	Safer technique with increased stability	[96]
Double emulsion crosslinking method	Cancer treatment	5-Fluorouracil	EE ≈ 0.60	Increased inhibition of cancer; controlled drug release; increased efficiency of entrapment	[97]
	Capillary hemangioma	Propranolol hydrochloride	EE ≥ 0.50	Minimal side effects; sustained drug release	[98]
Microemulsion method	Diabetes	Insulin	EE = 0.80%	Enhanced availability of the drug at the site (due to its interaction with the mucosal membrane of the intestine) and prolonged release of the drug; better compliance of oral delivery in patients	[99]
Crosslinking	Antimicrobial effects	Naringenin (NRG), quercetin (QE), and curcumin (CUR) conjugated with L-histidine and ZnO	LE varies from 0.89 to 0.92	Noticeable antimicrobial action against Trichophyton rubrum and Staphylococcus aureus strains because of the cumulative impact	[100]
	Breast cancer	Methotrexate	LE = 0.13	Sustainable drug release; improved drug loading efficacy	[101]
Droplet emulsion method	Glaucoma	Trimethylchitosan (TMC) and tetrandrine lipid NPs (TET-LNPs)-loaded carboxy-methylchitosan (CMC) or hydroxypropylchitosan (HPC)	LE ≥ 0.9	Increased bioavailability and retention time	[102]
Co-precipitation	Arthritis (rheumatoid)	Meloxicam	EE = 0.82	Lesser dosage frequency and toxicity	[103]
-	Antioxidant	Resveratrol	EE ≥ 0.90	Continuous release of the drug and enhanced storage and stability of the drug	[48]
Nano-precipitation	Parkinson’s Disease	Ropinirole hydrochloride coated with PGLA	LE = 0.05	Can cross the blood–brain barrier; hepatic metabolism; delivers the drug to the specific site of action	[49]

### 3.2. Development of Functionalized ChNPs

Chitosan possesses suitable functional groups that help with providing some specific properties to the polysaccharides. The structural and functional properties of chitosan become enhanced due to the presence of an amino group being present at the C-2 position. This group helps with providing the cationic nature, thus providing chitosan with various properties that include antimicrobial, wound healing, and mucoadhesive properties. The pKa value makes chitosan insoluble in water but soluble in various types of acidic solutions [104,105]. The mechanism of functionalization results in the development of N-modified, O-modified, and N,O-modified chitosan, thereby providing chitosan with a wide range of biological activities. The functionalization of chitosan via the use of quarternized N-alkyl or N-benzylchitosan or phosphorylation of the chitosan helps with the enhancement of the antimicrobial activity. O-modified chitosan is free to undergo the mechanism of N-modified derivatives when using H_2_SO_4_ or MeSO_3_H via the process of protonation of the amine group through the removal of hydroxyl group; this process is used for the purpose of protecting various types of hydroxyl groups [106]. Studies showed that cinnamaldehyde crosslinks with chitosan not only enhances its stability but also increases the antimicrobial efficacy of ChNPs [107,108]. It was also observed that ChNPs synthesized using the leaf extract of *Caltharanthus roseus* showed a size-dependent drug entrapment efficiency [109].

## 4. Inhibition of Biofilm Formation Using Functionalized Chitosan Nanoparticles

However, in order to target biofilm-associated chronic infections, medical devices, and food industries [110], the ChNPs must have the ability to block quorum sensing. It was revealed from various experimental observations that the positively charged ChNPs are usually loaded in Oxa or oxacillin and ChNP–DNase–Oxa or Deoxyribonuclease I [111]. The anti-biofilm activity is generally studied against the biofilm network formed by nosocomial bacterial species, such as *Staphylococcus aureus* and *Pseudomonas aeruginosa*. Biofilm structuring on silicone surfaces was checked and researched with the help of SEM or scanning electron microscopy [112]. Confocal laser scanning microscopy (CLSM) was used for looking upon alive or dead microorganisms inside the biofilm matrix, which revealed that ChNP–DNase–Oxa had a higher level of anti-biofilm activity than the Oxa-mixed nanoparticles, which is present without the ChNP–Oxa or the DNase and the summation of Oxa and DNase, which involves free Oxa [41,113]. Both the formation of new biofilms and the eradication of mature biofilms in vitro could be achieved with the help of the ChNP–DNase–Oxa. Actually, through the denaturation of eDNA, ChNP–DNase–Oxa can damage the biofilm matrix, decrease the width of the biofilm, and the number of viable cells on silicone. Back-to-back treating with the help of ChNP–DNase–Oxa over two days was seen to give a shocking and successful result of almost a 99% decrease in the biofilm [114]. Moreover, ChNP–DNase–Oxa was found to be effective against the biofilm of any type of normal and clinical strains of *Staphylococcus aureus* [113].

This shows the high potential and effectivity of nanoparticles for treating the infections associated with biofilms [115]. Attenuation of the signals of bacterial quorum sensing can inhibit infection and can also stop the generation of bacterial virulence. Many research works have been conducted and almost all of them showed that the natural compounds possess more effectiveness over artificially synthesized chemicals regarding their treatment of biofilms and establishing them as anti-quorum-sensing agents.

Especially, flavonoid compounds are highly efficient anti-microbial and antibiofilm compounds. However, due to the very low or no dissolution of the flavonoid molecules and the rare bioavailability, minimal application of flavonoids is found [116]. Experimental observations revealed that phytochemicals, when mixed with chitosan nanoparticles, significantly decreased the QS activity through the inactivation of AI molecules [117]. Kaempferol, a flavonoid, is known to possess high-anti-quorum-sensing activity [118]. The application of the kaempferol and chitosan nanoparticles was analyzed on the basis of their properties of hydrogen bonding, hydrodynamic diameter, antioxidant activity, and amorphous transformation. After this, the inhibition of the quorum-sensing molecules by the nanoparticles in a time-dependent pattern is usually studied [119]. This measurement is done in a violacein pigment with the help of a biosensor strain *Chromobacterium violaceum* CV026, which is again operated by an AI known as acylated homoserine lactone (AHL) [120]. Kaempferol-loaded sodium tripolyphosphate (TPP)on ChNPshave typical particle sizes and zeta potentials of 190 to 200 nm and +30 to +35 mV, respectively, and can be stored up to 30 days and still successfully inhibit the quorum-sensing molecules, namely, the violacein pigment, in *Chromobacterium violaceum* CV026 [121]. After the success of this method, attempts are being made to use it as a novel antimicrobial chemotherapy. In this process, the kaempferol-encapsulated chitosan nanoparticles play the role of a stable and effective quorum-sensing-dependent antimicrobial, antibacterial, and antibiofilm agent [122].

Quercetin (QUE), another flavonoid phytocompound that is found in many commonly used medicinal plants [123] holds strong potential for establishing itself as a QS-inhibiting agent against *Staphylococcus aureus*, *Pseudomonas aeruginosa*, etc. [124]. However, the effective laboratory application of quercetin alone has stopped because of its lesser solubility in physiological fluids [125]. Therefore, many research works convey a solubility increase strategy for quercetin, which is done in the form of an amorphous and stable complex of nanoparticles of quercetin and chitosan [126]. The preparation of this complex is done using an electrostatic method and it is performed to form a complex involving ionized quercetin components and oppositely charged ChNPs [127]. In optimal conditions, a quercetin and chitosan nanoparticle complex with a size of roughly 150 to 170 nm shows a payload of about 25 to 30% having 60 to 70% efficiency with a long storage ability. Due to the absence of any adverse side effects, the complex of quercetin and ChNPs can be used for various therapeutic purposes. Such a complex is found to be more effective in the inhibition of quorum sensing than quercetin alone. Although this complex could bring about the increased suppression of quorum-sensing-regulating genes, resulting in the haltingof swimming motility and formation of *Pseudomonas aeruginosa* biofilms, it could not suppress the formation of its virulence factor [46]. The prior inhibition of the production of the biofilm’s swimming motility using the quercetin and ChNPs complex revealed an almost five-fold increase in kinetic solubility [128].

A new type of ChNPsthat are dually crosslinked with genipin and sodium tripolyphosphate (TPP) display quorum quenching activity [129].

*Trans*-cinnamaldehyde (CA) is an intensively studied compound that was shown to inhibit QS activity by decreasing the DNA-binding ability of LuxR while inhibiting acyl-homoserine lactone production. In this work, chitosan-based nanocapsules laden with a high concentration of CA were applied to a transformed *E. coli* Top 10 strain fluorescence-based reporter [14,130].

## 5. Mechanism of QS Inhibition Using Functionalized Chitosan Nanoparticles

A nanocapsule is a shell made from a nontoxic polymer that encapsulates an inner liquid core at the nanoscale. These have many uses, including promising medical applications for drug delivery, food enhancement, nutraceuticals, and self-healing materials. The benefits of encapsulation methods are the protection of the drug and/or allied substances from the adverse environment, controlled release, and precision targeting. Hence, chitosan in the form of nanoparticles can exert its antibiofilm activity in a more targeted way. TPP-crosslinked nanoparticles (ionically crosslinked; IC-NPs) show considerable anti-quorum-sensing activity despite their inherent colloidal instability in microbiological media.

It was found that nanocapsules can interact with bacteria via electrostatic interaction, thus effectively delivering the quorum-quenching compound CA to the bacteria. The electrostatic adsorption of the chitosan-coated nanocapsules to the bacterial cell envelope is the mechanism that underpins the observed enhancement of the QS inhibition activity [131].

The polycationic groups in organic nanoparticles that are used for antimicrobial activity cause cell damage, perhaps via an ion exchange interaction between bacteria and charged polymer surfaces, resulting in the disruption of cellular membranes [132]. Polycationic nanoparticles can enter into cells via endocytosis, followed by the formation of nanoscale membrane holes, which leads to a final membrane translocation. [133]. The mechanism of interaction of nanoparticles on the cell surface was also reported in terms of the adsorption and penetration (or disruption) of cell membranes, triggering NP-mediated toxicity. This may include steps such as nanoparticle adhesion at the membrane/water interface, passive membrane translocation, membrane restructuring and leakage, and adhesive lipid extraction [134]. Nanoparticle translocation into a cell is observed to occur via the outer wrapping, followed by free translocation and inner attachment and embedment [135]. The adsorption of NPs leads to cell wall depolarization, inducing cellular toxicity and degradation, which allows ions to enter the cytosol. Sometimes, NPs cause irregular pits on the cell wall surface, enabling ions to enter the cell [136]. The polysaccharides of EPS interact with SO_4_ groups of functionalized polystyrene NPs via hydrophobic complexation, which disrupts bacterial biofilm formation [137].

Quaternary ammonium chitosan NPs can produce long cationic polymer chains that penetrate the cell membrane and can induce ion exchange, which disrupts biofilms [138].

**Figure 2:**
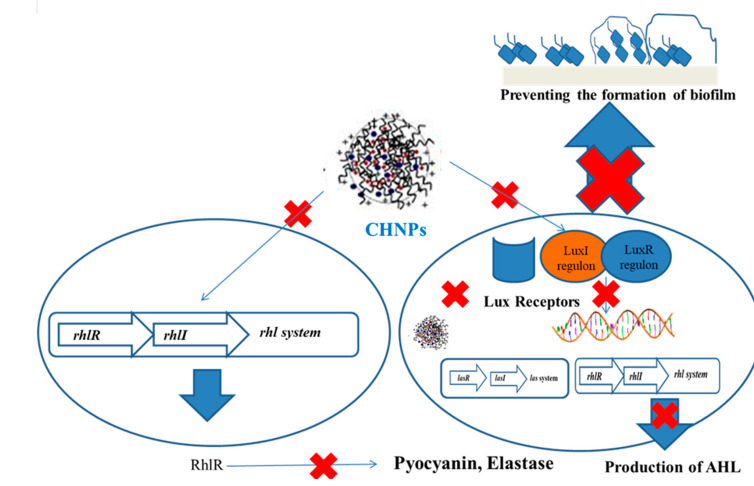
Mechanism of inhibition of biofilm by ChNPs.

The positive surface of QAS ciprofloxacin-loaded nanochitosan-coated Ti implants disintegrates the negatively charged bacteria, followed by the release of ciprofloxacin, which inhibits enzymes, including DNA gyrase, and topoisomerase causes bacterial disruption. Free radicals interact with endogenous molecular oxygen to produce ROS, superoxide hydroxyl radicals, and hydrogen peroxide, which damages the bacteria membrane integrity and causes irreparable bacteria lysis [139]. Quaternized-chitosan-loaded Ag NPs release Ag ions that disintegrate the bacteria and inhibit biofilm development [140] (Figure 2).

## 6. Conclusions

After surface functionalization with various bioactive compounds, chitosan nanoparticles become well equipped for quorum quenching. The success of biofilm eradication lies with the precise obstruction of the transmission of signal molecules for cell-to-cell communication. More research is warranted to find out potent bioactive compounds that can be used for the functionalization of chitosan nanoparticles for various successful therapeutic applications.

## Figures and Tables

**Table 1:** Quorum-sensing (QS) systems of selected Gram-negative bacteria.

SL No.	Bacterial Organism Name	Quorum-Sensing Molecules	Genes	Receptors	References
1.	*Chromobacterium violaceum*	C12-HSL	N.A.	N.A.	[34]
		N.A.	N.A.	SdiA	[35]
		AI-2	*LuxS*	LsrB	[16,36]
2.	*Pseudomonas aeruginosa*	C4-HSL	*RhlI*	RhlR	[37]
		3-oxo-C12-HSL	*LasI*	LasR	[38,39]
		3-oxo-C12-HSL	*NA*	QscR	[3,40]
		PQS, HHQ	*PqsABCD, PqsH*	PqsR	[41]
3.	*Staphylococcus aureus*	3-hydroxy-C4-HSL	*LuxM*	LuxN	[3,40]
		AI-2	*LuxS*	LuxP	[42]
		CAI-1	*CqsA*	CqsS	[43]
4.	*Acinetobacter baumannii*	3-hydroxy-C12-HSL	*AbaI*	AbaR	[44]
5.	*Escherichia coli*	3-oxo-C8-HSL	N.A.	SdiA	[27,35]
		AI-2	*LuxS*	LsrB	[37,45,46]
		AI-3/Epinephrine/Norepinephrine	N.A.	QseC	[47]
6.	*Klebsiella pneumoniae*	C8-HSL	N.A.	N.A.	[15,36]
		C12-HSL	N.A.	N.A.	[27]
		AI-2	*LuxS*	LsrB	[48,49]

## Data Availability

Not applicable.
